# Revision Total Hip Arthroplasty Using a Custom Monoflange Acetabular Component for Severe Bone Loss and Pelvic Discontinuity Due to Acetabular Component Loosening: A Case Report

**DOI:** 10.7759/cureus.71167

**Published:** 2024-10-09

**Authors:** Andrzej Bałoniak, Tomasz Markiewicz, Sławomir Michalak, Mateusz Pochylski, Jan Stępka, Julia Woźna, Łukasz Łapaj

**Affiliations:** 1 Department of General Orthopedics, Orthopedic Oncology and Traumatology, Poznan University of Medical Sciences, Poznań, POL

**Keywords:** cmac, custom monoflange acetabular component, implants, revision, total hip arthroplasty

## Abstract

Revision total hip arthroplasty using custom monoflange acetabular components (CMACs) in cases of severe bone loss is becoming an increasingly preferred alternative to traditional implants, which may not provide stable fixation in such cases. However, this procedure also carries a higher risk of infection and implant loosening. This case report discusses a 67-year-old female patient who presented with old acetabular component loosening and significant pelvic bone loss. A 3D model of the pelvis was created based on computed tomography, enabling the design of a personalized monoflange acetabular component. The revision surgery was performed using a posterolateral approach, and the implant was successfully secured. After six months, the patient reported no complications or pain. It seems that CMACs offer a promising alternative for patients presenting with chronic implant loosening and severe bone stock loss.

## Introduction

Severe bone stock loss due to chronic loose acetabular components is a very serious late complication of total hip arthroplasty (THA) that requires challenging revision surgery. Custom-made implants are a further treatment option when a severe osseous defect of hemipelvis occurs and there is no suitable standard implant available. Personalized implants are nothing new, and there are several possibilities for reconstruction of the acetabulum in the hip joint ​[1,2]​. The undeniable advantage of custom implants is the ability to adjust the implant orientation and center of rotation (COR) to the patient's native anatomy. However, revision surgeries using custom implants are associated with a higher risk of infection, implant loosening, and consequently, an increased risk of further revisions ​[3,4]​. Custom monoflange acetabular components (CMACs) are a viable alternative, particularly for patients with significant loss of supportive bone in the acetabular roof and posterior wall. However, their implantation is technically challenging, with implant positioning being a particularly difficult stage. In this case report, we discuss a patient with severe bone loss who was treated with revision total hip arthroplasty (rTHA) using a CMAC.

## Case presentation

In 2022, a 67-year-old woman was admitted to the Department of General Orthopedics, Orthopedic Oncology and Trauma Surgery, Wiktor Dega Hospital, for elective rTHA of the right hip due to severe bone stock loss and pelvic discontinuity. Primary THA was conducted in 2007. In 2016, a dislocation was diagnosed; however, the patient did not pursue treatment for the condition. In 2018, the patient started to feel pain in the right hip. In 2020, the patient was admitted for the rTHA; however, due to personal problems, the patient left the clinic on demand. In 2022, the patient returned to the hospital.

**Figure 1:**
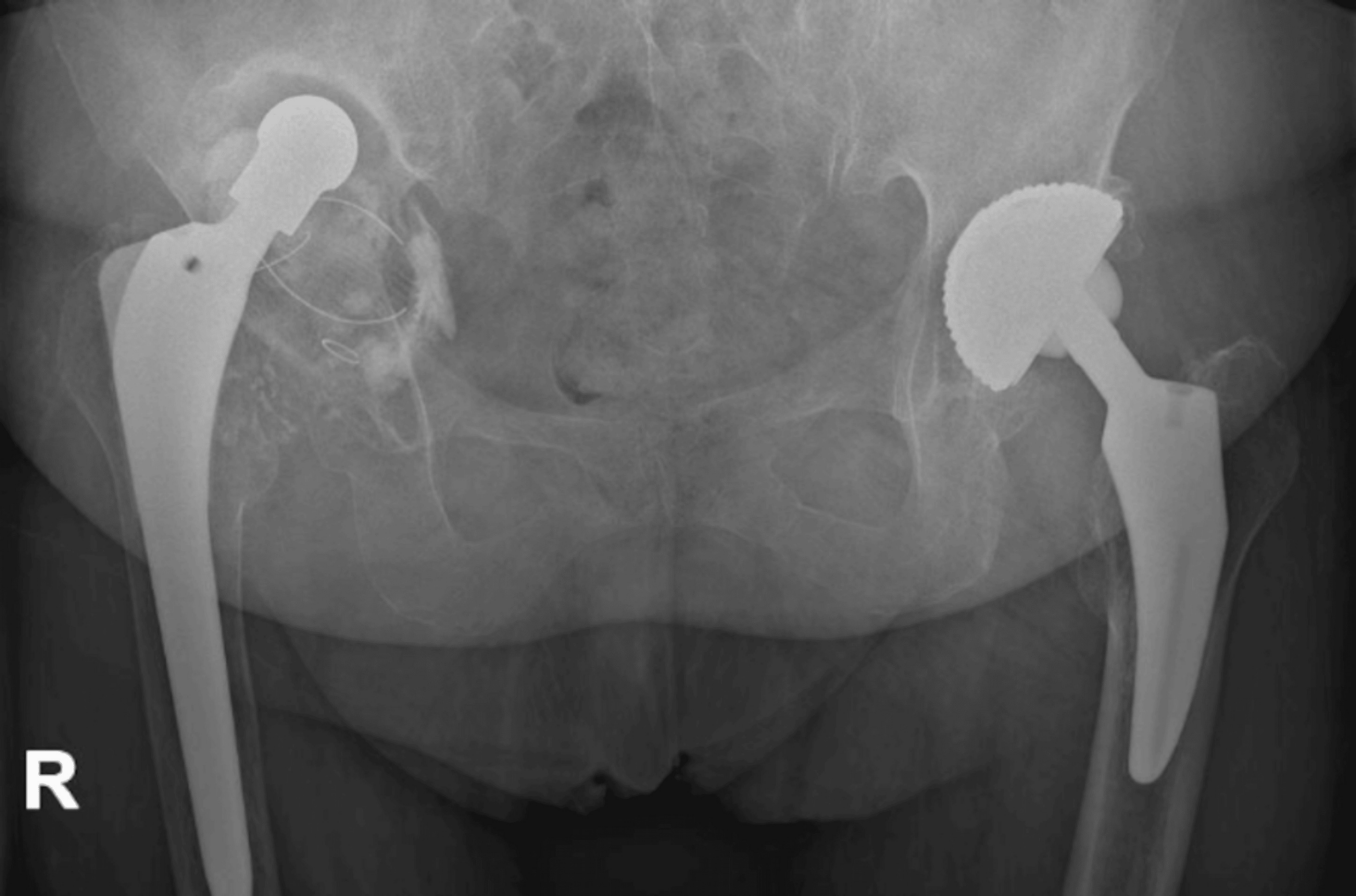
RTG before the surgery from April 2022. Visible loosening of the right hip implant with massive destruction of the pelvic bone.

**Figure 2:**
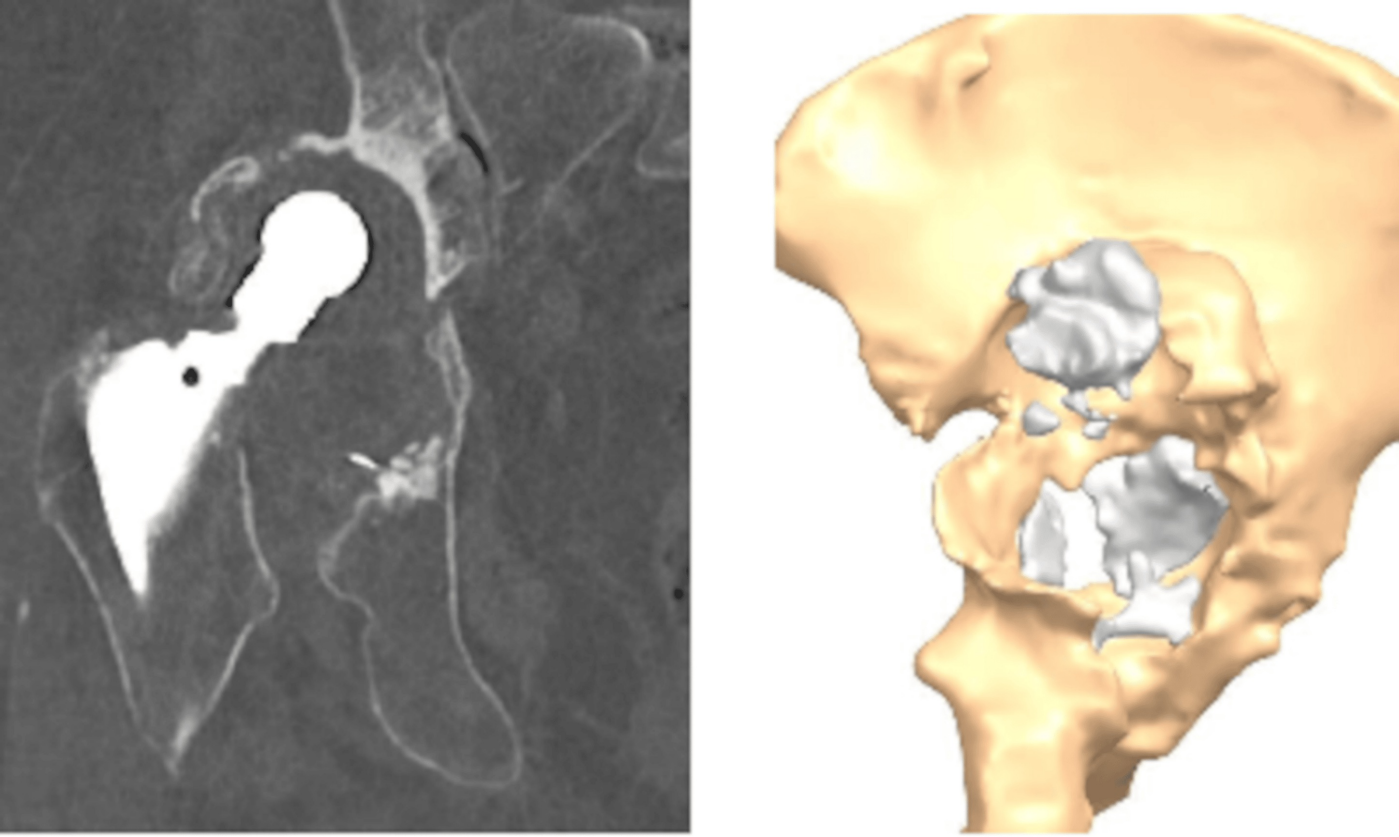
Thin-layer computed tomography with 3D reconstruction from February 14, 2022. CT scan quality leading to uncertainty in 3D reconstruction.

**Figure 3:**
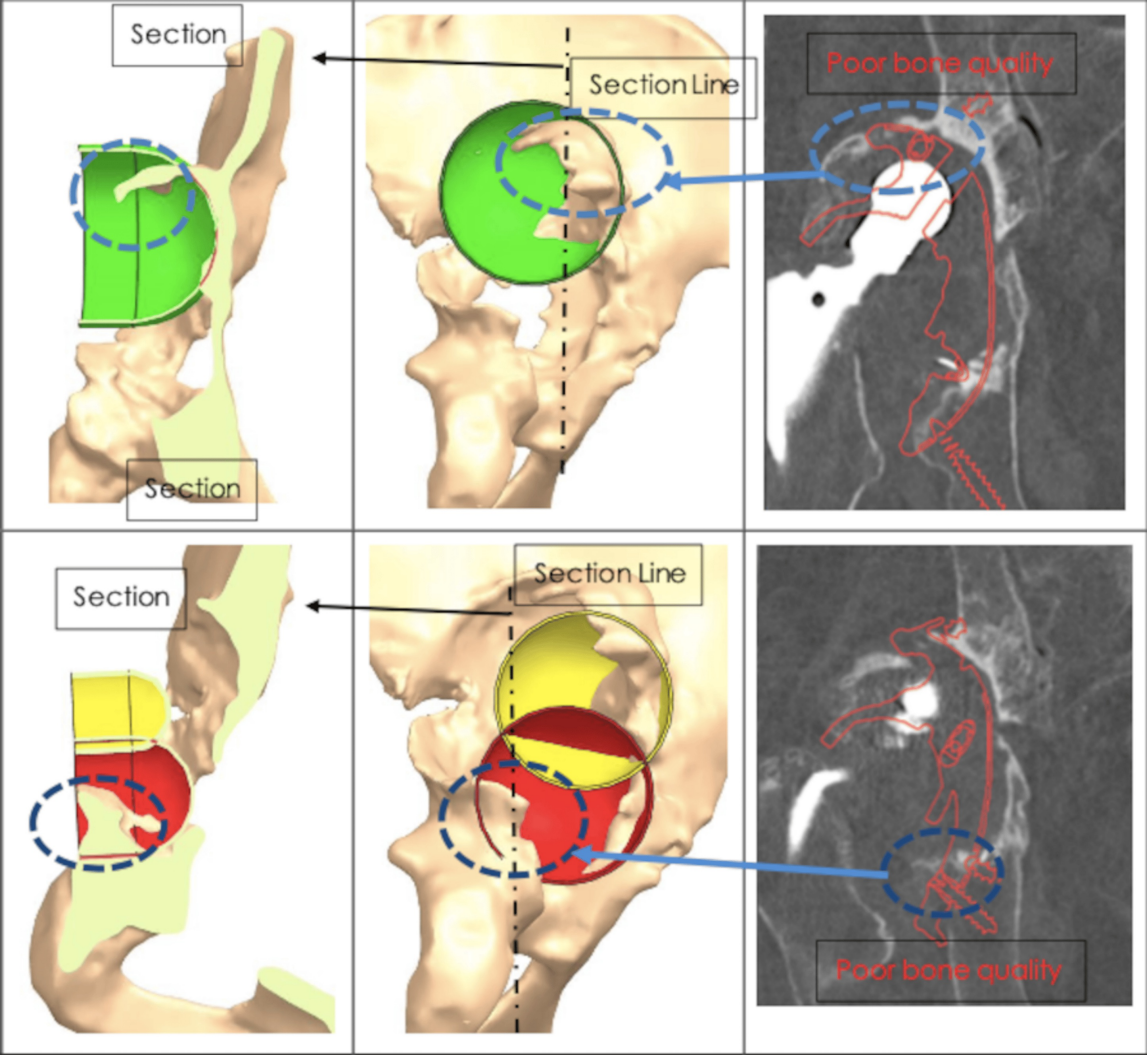
Planned reamings. The 68mm diameter reaming (green) was designed to increase contact between the implant and native bone on the cranial side and remove poor-quality bone, ensuring cup stability, particularly in the load-bearing direction. The two 55mm reamings (red and yellow) improved contact on the caudal side and were performed with the goal of limiting leg length discrepancy to less than 30mm.

**Figure 4:**
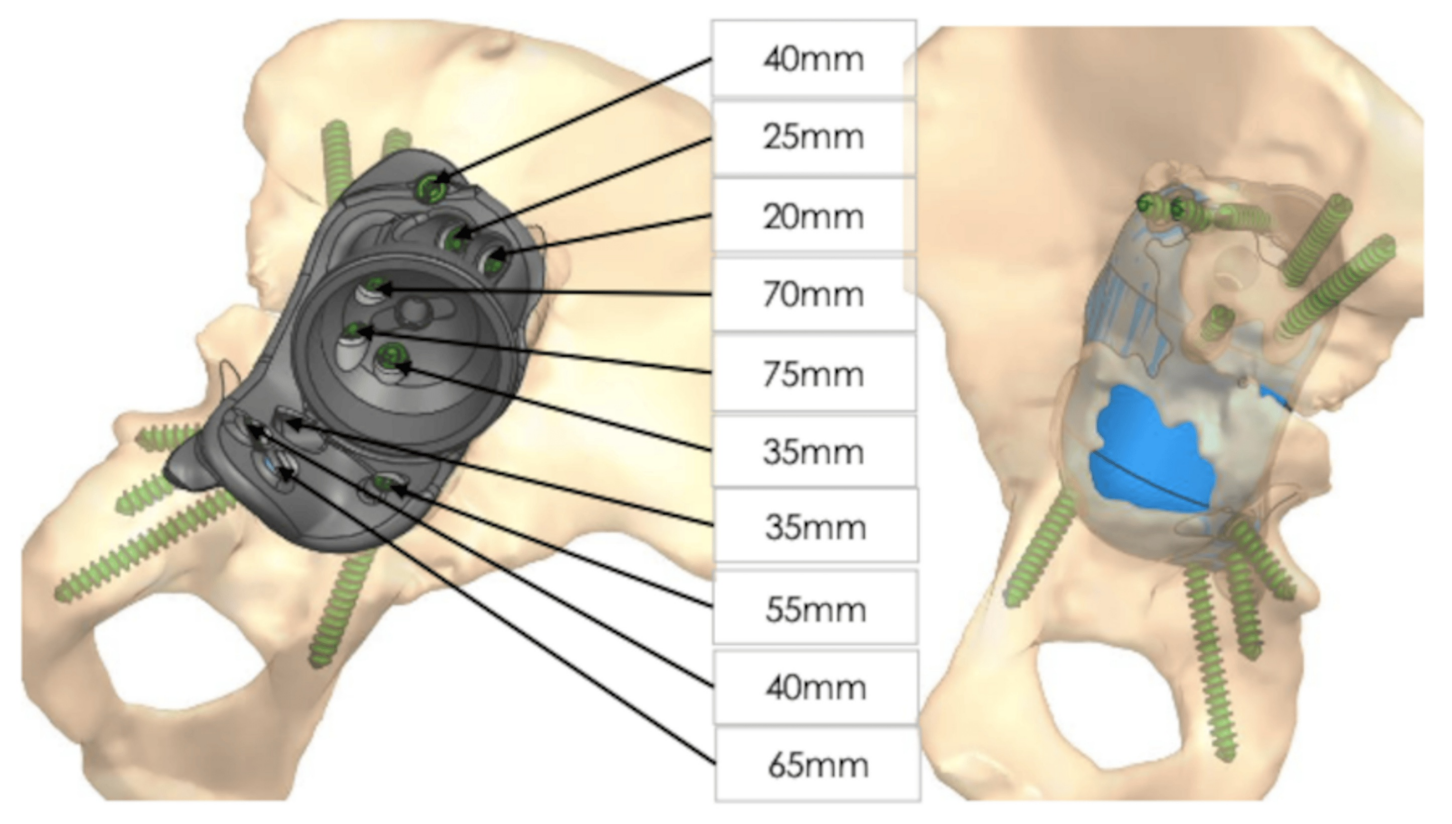
Custom-made acetabular component with one cranial augment and with one caudal augment. One millimeter of press fit between the cup and the prepared bone. Fixation with 6.5mm diameter compressive bone screws. Three screws in the ischium ramus and one screw in the pubic ramus, one screw in the iliac flange, and five screws in the ileum. The blue region is the area of bone loss.

Severe bone stock loss and pelvic discontinuity (Paprosky type 4 defect) and erosion of medial and posterior wall along with dislocated hip prosthesis are seen in Figure 1. The patient could not walk without a walker. A decision was made to fabricate CMAC. The patient underwent a thin-layer computed tomography scan with 3D reconstruction (Figure 2). Three planned reamings were established to remove poor-quality bone (Figure 3). The implants were planned to be secured using specially selected screws of specific length and trajectory, placed in areas of good bone quality (Figure 4). In this particular case, the engineers followed the surgeons' recommendations and designed an implant with minimal flanges. Since the preparatory stage required three reamings and the initial trial placement to determine orientation, there was a significant risk of misaligning the final implant. As a result, the implant was designed with minimal flanges so that primary press-fit and screw fixation would serve as the main fixation methods, rather than the three external flanges commonly used in most designs.

According to the Visual Analogue Scale (VAS), the patient reported a pain intensity of eight out of 10 on the day before the revision surgery. The Western Ontario and McMaster Universities Osteoarthritis Index (WOMAC) score was 44.8% (43/96 points) prior to the surgery. In October 2022, a revision total hip arthroplasty was performed using a posterolateral approach. The surgery lasted four hours.

**Figure 5:**
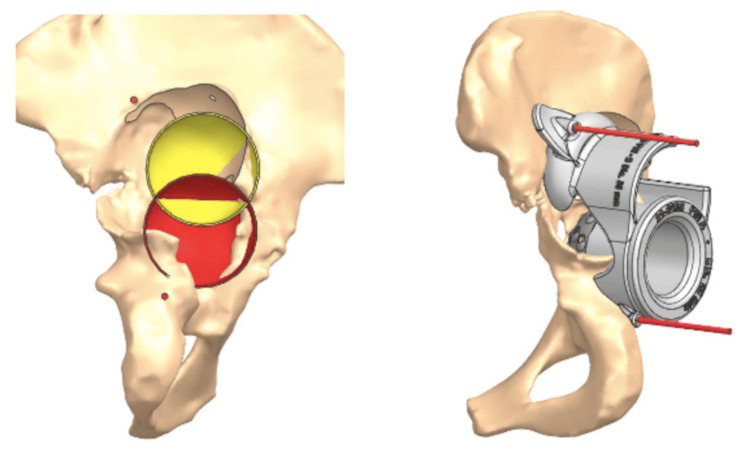
Positioning the reamer stopper to verify the caudal preparation. The red and yellow regions indicate two 55mm reamings.

**Figure 6:**
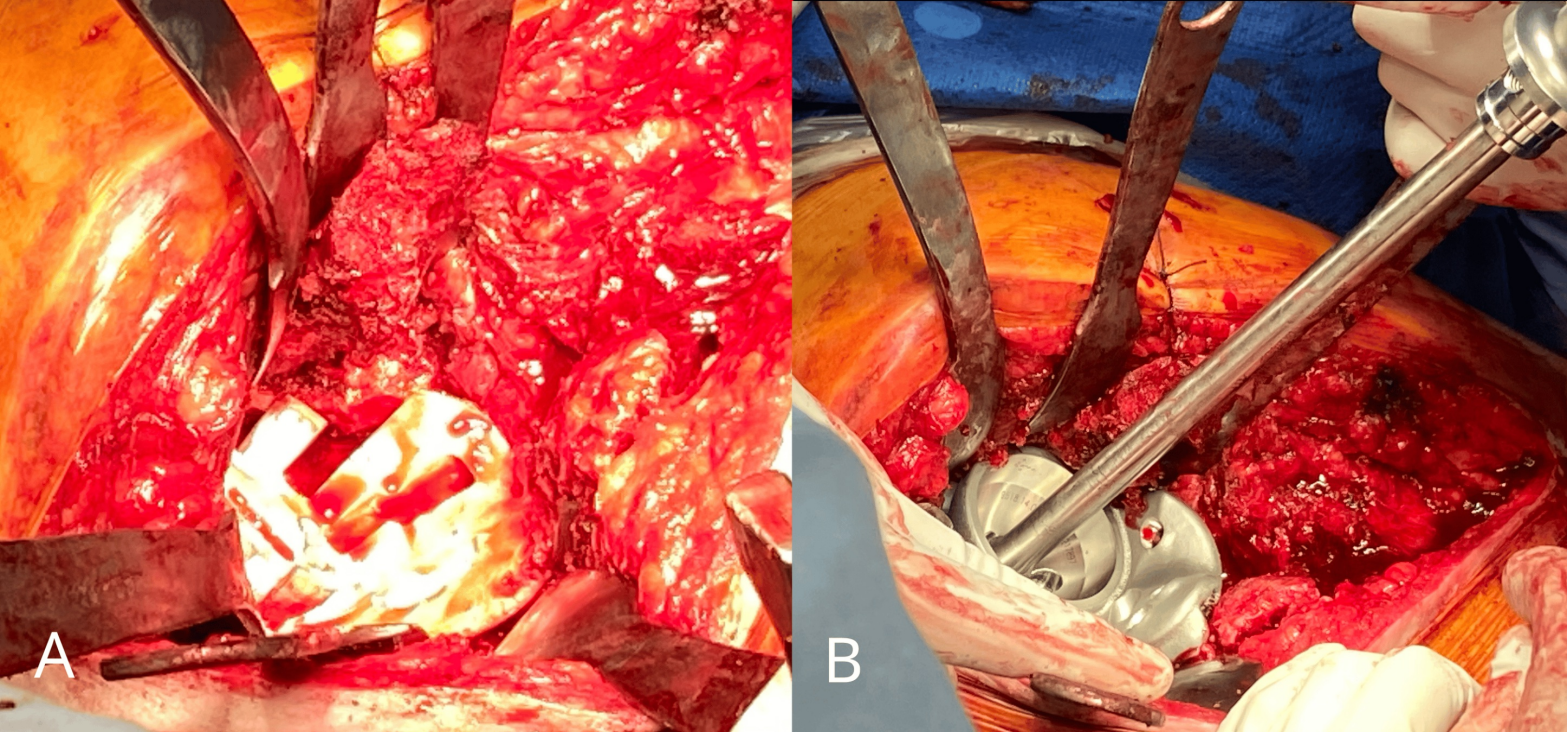
Trial fitting and CMAC placement. A. Trial fitting of the plastic prototype. B. CMAC placement with insertion of the bone screws.

Initially, the previous implant and cement were removed. Due to difficulty in reorienting the femur for adequate acetabular exposure, a decision was made to perform a trochanteric osteotomy to mobilize the femur. Once acetabular exposure was improved, extensive debridement and removal of osteophytes and periprosthetic calcifications were carried out. Plastic trial models were then utilized to identify anatomical landmarks and determine the extent of bone removal required. This stage was challenging due to the presence of periprosthetic ossifications and osteophytes that needed to be excised. The surgeon began reaming with smaller reamers, followed by a 68mm reamer used freehand to prepare the cranial spherical cavity. Next, the 55mm reamer head was fitted with a reamer plug, and a reamer stopper was positioned to check the caudal preparation cranially, posteriorly, and along the most proximal side of the ischium (Figure 5). The 55mm reamer was then used to prepare the spherical caudal cavity. A flangeless trial was employed to assess proper reaming and implant fit. The surgical team then proceeded to place the CMAC (Figures 6A, 6B). A drilling guide was used to drill in the templated direction, and 6.5mm compressive bone screws were inserted, starting with the internal screws to enhance implant-bone compression. The final implants were successfully secured, including a Size L Spacer. Finally, the greater trochanter osteotomy was stabilized using a Stryker plate and two metal wires.

**Figure 7:**
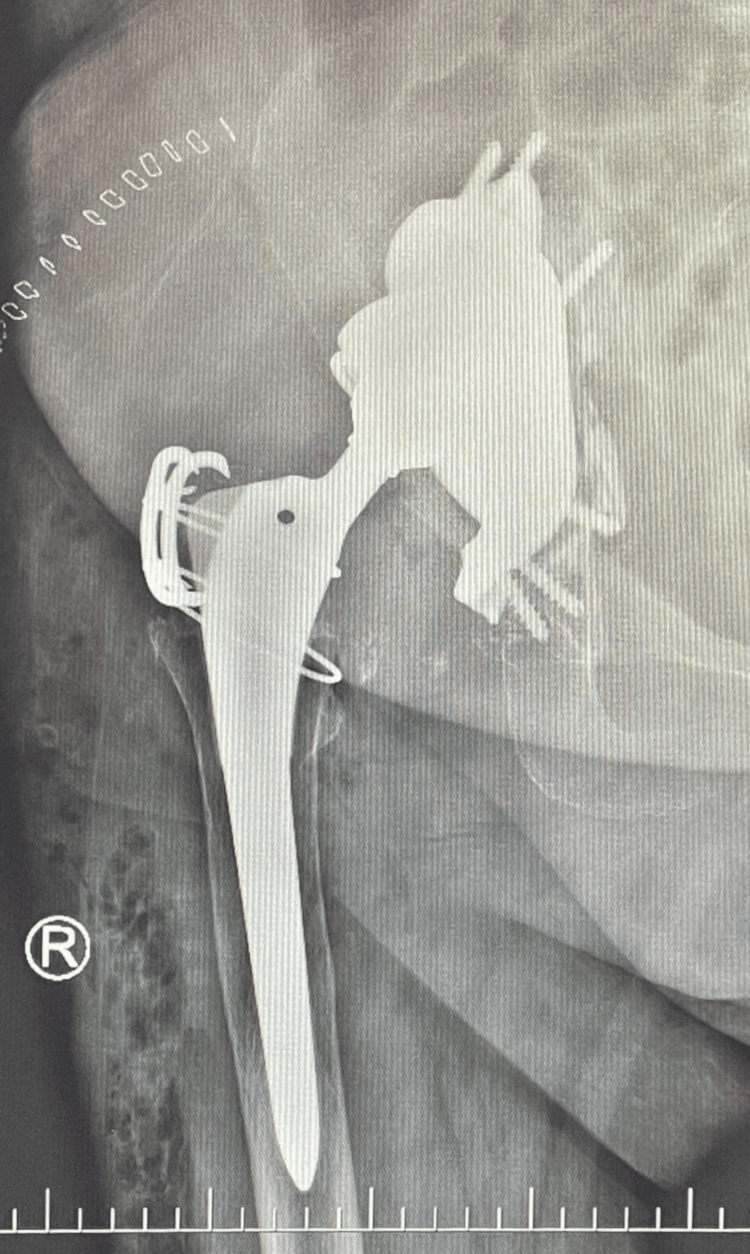
Final joint reconstruction.

Postoperatively, the patient's condition was critical, necessitating a transfusion of red blood cell concentrate and fluids. No complications were observed in the early postoperative period. A postoperative radiograph confirmed successful joint reconstruction and restoration of center of rotation (COR) (Figure 7). At the six-month follow-up, no complications such as implant loosening, periprosthetic infection, dislocation, periprosthetic fracture, or osteolysis were noted. The VAS score had decreased to 0, the WOMAC score was also 0% (0/96 points), and the patient's satisfaction with the surgery was rated 5/5 at that time.

## Discussion

CMACs are increasingly used in revision THA, especially in cases of severe bone loss and pelvic discontinuity. This case demonstrates the effective use of a CMAC to address substantial bone loss and prosthesis dislocation, where conventional implants were inadequate.

Two main challenges arise in such cases: achieving adequate fixation and restoring the center of rotation (COR) and ensuring accurate trial component positioning during reaming. Misalignment can lead to screw misplacement, pelvic perforation, and compromised fixation. While traditional modular systems often fail in cases of pelvic discontinuity, custom-made components are tailored to the patient’s bone defect. Although flanges offer structural support from an engineering perspective, they can complicate surgery. Despite the lack of flanges, the postoperative stability and fixation achieved in this case suggest that CMACs provide effective COR restoration, at least comparable to custom-made triflange acetabular components (CTACs) ​[5]​.

In our study, the patient’s VAS was 0, and the WOMAC score was 0%, indicating the absence of pain and good functional restoration, which is uncommon in the postoperative period. Most studies indicate that patients undergoing rTHA with CMACs tend to experience mild pain and some degree of functional limitation in the operated hip during the later postoperative period ​[6,7]​.

The future of rTHA for patients with severe bone stock loss is closely linked to advancements in personalized implants, especially 3D printing technology. These innovations allow for precise anatomical replication, leading to better implant fit and potentially longer-lasting results. However, custom implants are significantly more expensive than standard options and require more extensive surgery and longer operating times. In our study, surgery lasted around 240 minutes, consistent with other reports ​[8,9]​. Larger implant sizes require more extensive soft tissue dissection. Prolonged exposure of a wide surgical field increases the risk of infection, while the large size of the implant carries a higher risk of loosening. This raises concerns regarding the long-term survival of custom implants. Recent meta-analysis results assessing the performance of custom-made triflange acetabular components (CTACs) have demonstrated a failure rate of 12% at a mean follow-up of 46.9 months ​[10]​ . This represents a significantly lower failure rate compared to the study by Fröschen et al., which reported implant survival rates of 82.7% at three years and 77% at five years following revision total hip arthroplasty with the use of custom monoflange acetabular components (CMACs) ​[8]​. In the study conducted by Walter et al., there were no differences in implant survival rates between CMACs and CTACs ​[6]​. Our case also demonstrates that in cases of cup loosening with severe bony defects and pelvic discontinuity custom implants without flanges can significantly improve short-term patient outcomes in complex revision cases involving severe bone loss and pelvic discontinuity. However, it lacks long-term follow-up data, limiting conclusions about sustained outcomes over time.

## Conclusions

Revision surgery using custom monoflange acetabular components (CMACs) in patients with severe bone stock loss may enhance postoperative quality of life and facilitate their return to normal activities. A customized implant is a viable alternative in rare cases, such as chronic hip prosthesis dislocations with severe bone stock loss and pelvic discontinuity. The long-term clinical outcomes of revision total hip arthroplasty using custom-made components have yet to be fully explored.
